# Comprehensive analysis of clinical outcomes, infectious complications and microbiological data in newly diagnosed multiple myeloma patients: a retrospective observational study of 92 subjects

**DOI:** 10.1007/s10238-024-01411-2

**Published:** 2024-06-27

**Authors:** Vanessa Desantis, Paola Borrelli, Teresa Panebianco, Antonio Fusillo, Donatello Bochicchio, Angelo Solito, Fabrizio Pappagallo, Antonella Mascolo, Anna Ancona, Sebastiano Cicco, Claudio Cerchione, Alessandra Romano, Monica Montagnani, Roberto Ria, Angelo Vacca, Antonio Giovanni Solimando

**Affiliations:** 1 Department of Precision and Regenerative Medicine and Ionian Area (DiMePRe-J), Section of Pharmacology, University of Bari “Aldo Moro” Medical School, Bari, Italy; 2 Department of Medical, Oral and Biotechnological Sciences, Laboratory of Biostatistics, University “G. d’Annunzio” Chieti-Pescara, Chieti, Italy; 3 Department of Precision and Regenerative Medicine and Ionian Area (DiMePRe-J), Unit of Internal Medicine “Guido Baccelli”, University of Bari “Aldo Moro” Medical School, Bari, Italy; 4 Department of Hematology, IRCCS Istituto Scientifico Romagnolo per lo Studio e la Cura dei Tumori (IRST), Meldola, Forlì-Cesena Italy; 5 Department of General Surgery and Medical-Surgical Specialties, Hematology Section, University of Catania, Catania, Italy

**Keywords:** Multiple myeloma, Sepsis, Septic shock, Listeria monocytogenes, Anti-CD38 antibody therapy

## Abstract

Patients with multiple myeloma (MM) have an increased risk of sepsis due to underlying disease- and treatment-related immunosuppression. However, data on sepsis incidence, causative pathogens, and impact on outcomes in newly diagnosed MM (NDMM) are limited. We conducted a retrospective observational study of 92 NDMM patients who developed sepsis between 2022 and 2023 at a tertiary care center in Italy. Patient characteristics, sepsis criteria [Quick Sequential Organ Failure Assessment, Systemic Inflammatory Response Syndrome (SIRS)], microbiology results, and associations with progression-free survival (PFS) were analyzed. In this cohort of 92 critically-ill patients, pathogenic organisms were identified via microbiological culture in 74 cases. However, among the remaining 18 culture-negative patients, 9 exhibited a SIRS score of 2 and another 9 had a SIRS score of 4, suggestive of a clinical presentation consistent with sepsis despite negative cultures. Common comorbidities included renal failure (60%), anemia (71%), and bone disease (83%). Gram-negative (28%) and Gram-positive (23%) bacteria were frequent causative organisms, along with fungi (20%). Cox Univariate analyses for PFS showed statically significant HR in patients with albumin ≥ 3.5 vs < 3.5 (HR = 5.04, *p* < 0.001), Karnofsky performance status ≥ 80 vs < 80 (HR = 2.01, *p* = 0.002), and early-stage vs late-stage disease by International Staging System (HR = 4.76 and HR = 12.52, both *p* < 0.001) and Revised International Staging System (R-ISS III vs R-ISS I, HR = 7.38, *p* < 0.001). Sepsis is common in NDMM and associated with poor outcomes. Risk stratification incorporating sepsis severity, comorbidities, and disease stage may help guide preventive strategies and optimize MM management.

**Supplementary Information:**

The online version contains supplementary material available at 10.1007/s10238-024-01411-2.

## Introduction

Sepsis and septic shock pose significant challenges in patients with newly diagnosed multiple myeloma (NDMM) [1], leading to substantial morbidity and mortality [2]. The management of sepsis in this patient population is complex and requires precise identification and prompt intervention. Multiple myeloma (MM) is characterized by the clonal proliferation of plasma cells (PCs) in the bone marrow (BM), leading to the production of monoclonal immunoglobulins [3]. The disease not only compromises the normal immune response but also impairs the production of functional immunoglobulins, rendering patients susceptible to infections [4]. The presence of monoclonal proteins and the suppression of polyclonal immunoglobulins, particularly IgG and IgA subclasses, contribute to immune dysfunction and increase the risk of severe infections, including sepsis [5].

Sepsis is a systemic inflammatory response syndrome caused by an infection that can progress to septic shock, resulting in organ dysfunction and high mortality rates [6]. In MM patients, sepsis represents a major challenge due to the complex interplay between the disease, immunosuppression, and the emergence of antimicrobial resistance. The compromised immune system, coupled with the impaired humoral immune response, predisposes MM patients to infections, which can rapidly escalate to sepsis [7]. Furthermore, the presence of BM disease, renal failure, and anemia, characteristic features of MM, further exacerbates the severity and complications of sepsis in this patient population [8]. Despite advancements in the management of MM, sepsis remains a significant unmet need. The diagnosis of sepsis in MM patients is often challenging due to the overlapping clinical manifestations of the underlying disease and infection. Additionally, there is a lack of consensus on the optimal sepsis diagnostic criteria and scoring systems in MM patients, leading to variability in clinical practice and potential delays in appropriate treatment initiation. Consequently, the timely identification and management of sepsis in MM patients are crucial to improve clinical outcomes and reduce mortality rates [9]. Gram-negative bacteria, including *Klebsiella pneumoniae* and *Pseudomonas aeruginosa*, are frequently implicated in respiratory tract infections, while gram-positive bacteria, such as *Staphylococcus aureus* and *Streptococcus pneumoniae*, contribute to the infectious burden. Fungal infections, predominantly *Candida* species, and rare pathogens like *Listeria monocytogenes* further complicate the microbiological landscape of sepsis in MM patients, necessitating tailored antimicrobial strategies [10]. Addressing the unmet need in sepsis management in MM patients requires a comprehensive understanding of the clinical characteristics, immune dysregulation, and microbiological factors contributing to the development and outcomes of sepsis.

This manuscript presents a comprehensive analysis of clinical data derived from a cohort of 92 NDMM patients who developed sepsis or septic shock. Here we investigated the impact of Sepsis-3 criteria and sepsis scoring systems, including quick Sequential Organ Failure Assessment (qSOFA) [6, 11] and Sistemic Inflammatory Response Syndrome (SIRS) [12], on the diagnosis, clinical outcomes, immune profiling, and microbiological data in NDMM patients. Additionally, the study explores MM-specific features, including the International Staging System (ISS) staging, the Hypercalcemia, Renal dysfunction, Anemia, Bone disease (CRAB criteria), and the 60% PCs, serum-free light chain involvement and greater than one lesion detected by magnetic resonance (SLiM criteria), to elucidate their association with sepsis development and severity [13]. Furthermore, the analysis of therapy modalities, such as autologous stem cell transplant (ASCT) versus non-ASCT approaches, triplet regimens, and antibody therapies (e.g., anti-CD38 monoclonal antibodies) [14, 15], will shed light on their influence on sepsis occurrence and outcomes.

Understanding the impact of these treatment modalities on the immune system and susceptibility to infections is crucial for optimizing therapeutic approaches and minimizing the risk of sepsis in MM patients. By providing insights into the clinical, immunological, and microbiological aspects of sepsis in NDMM patients, this study points to developing tailored strategies for the identification, prevention, and management of sepsis in this vulnerable patient population [16]. The findings have the potential to improve patient outcomes, reduce the burden of sepsis, and ultimately enhance the overall management of MM outcomes [17].

## Materials and methods

### Patients

We conducted a retrospective observational study of 92 NDMM patients who developed sepsis between 2022 and 2023 at a tertiary care center in Italy. The study was conducted in conformity with the Good Clinical Practice Guidelines of the Italian Ministry of Health and the ethical guidelines of the Declaration of Helsinki (as revised and amended in 2004), with the approval of the Ethics Committee of the University of Bari Medical School (study n° 7411, prot. n° 0073322, 26/08/2022). All enrolled patients provided their informed consent.

### Clinical, laboratory evaluation and *sepsis* criteria

Progression-free survival (PFS) was calculated from the date of diagnosis to the event, namely myeloma disease progression or death. The median follow-up for PFS calculation was 30.16 months (interquartile range -IQR- 17.17–43.61). Data were collected by thoroughly reviewing the medical records of the included patients. Relevant information regarding demographics, diagnostic criteria, sepsis scores, microbiological analysis and treatment modalities were extracted and recorded.

The Sepsis criteria (qSOFA, SIRS) were utilized to diagnose sepsis in the patient cohort. The application of these criteria led to the identification of sepsis in 73 patients. Additionally, 19 patients were diagnosed with septic shock. No other septic events requiring hospitalization were observed for enrolled patients during follow-up.

Microbiological analysis was performed to identify the causative pathogens responsible for sepsis in the patient population.

### Statistical analysis

Descriptive analysis was carried out using means and standard deviation or median and IQR for the quantitative variables and percentages values for the qualitative ones. Normality distribution for quantitative variables was assessed by the Shapiro–Wilk. Univariate comparisons were investigated using the Pearson chi-square test or the Fisher’s exact test for categorical data. Survival analysis was performed by applying the Kaplan–Meier estimator and log-rank test for equality of survivor functions. The association with clinical features was analyzed with the Cox model of proportional hazards (hazard ratio -HR- and 95% CI), and the applicability assumption was evaluated by the Schoenfeld test. Statistical significance was taken at the ≤ 0.05 level. All analyses were performed using STATA software 18.0 MP Edition (StataCorp, College Station, Texas, USA).

## Results

### Characteristics of MM patients with *sepsis*

Ninety-two patients (53% male and 47% female) with NDMM, fulfilling the International Myeloma Working Group [18] (IMWG2014 diagnostic criteria for symptomatic MM), who developed sepsis or septic shock were enrolled. The patients had a median age of 62.5 (IQR 53–74) years. Among the 92 NDMM patients, a significant proportion of individuals developed sepsis or septic shock. In terms of MM treatment modalities, a notable proportion of patients underwent ASCT, highlighting its importance as a therapeutic approach in eligible MM patients. For patients who did not undergo ASCT, triplet regimens consisting of proteasome inhibitors, immunomodulatory drugs, and corticosteroids were commonly administered, reflecting the current standard of care for non-ASCT patients (63%) [19]. Specifically, across the entire cohort 34 received ASCT, 58 did not. The median value of time interval over the entire cohort was -30 (IQR -302;-21). There is no difference in median values of time interval (days) from the treatment initiation and the sepsis event when stratified for the qSOFA (*p* = 0.954), SIRS (*p* = 0.843). ASCT impact was also deemed neither statistically nor clinically significant in our cohort (*p* = 0.068) (Supplementary Table 1). Additionally, a proportion of patients received anti-CD38 monoclonal antibody therapy, which has shown efficacy in MM treatment (35%) (Table 1). No patients treated with CAR-T were recruited and all patients had different combination regimens (Dara-based or not-Dara-based) in the combination regimen as by protocols approved by the regulatory agency in Europe [20] (Supplementary Table 2).

**Table 1:** Patients' baseline characteristics and previous treatments

Characteristics	*N* (%)
Sex	
Male	49 (53)
Female	43 (47)
Age	
Median (IQR)	62.5 (53–74)
Heavy chain	
IgG	46 (50)
IgA	40 (43)
Micromolecular	6 (7)
Light chain	
κ	5 (83)
λ	1 (17)
Cytogenetic risk	
Yes	44 (48)
No	48 (52)
First line	
ASCT recipient	34 (37)
non-ASCT recipient	58 (63)
Treatment regimen	
Dara-based	32 (35)
non-Dara-based	60 (65)
Response to therapy	
Optimal (sCR, CR, VGPR)	46 (50)
Suboptimal (PR, SD)	46 (50)

*ASCT* Autologous stem cell transplantation; *DARA* Daratumumab; *sCR* Stringent complete response; *CR* Complete response; *VGPR* Very good partial response; *PR* Partial response; *SD* Stable disease

All the patients received antimicrobial prophylaxis with trimetoprim/sulphamethoxazole and acyclovir as by recommendations [20].

Applying the Sepsis criteria, sepsis was diagnosed in 74 patients (80%), while 19 patients had septic shock (21%), indicating the severity of their condition. For the sepsis group the qSOFA score is 3 in 82% of patients, and the SIRS score is 4 in 80% of patients, reflecting the overall disease burden and organ dysfunction (Table 2).

**Table 2:** Characteristics of MM Patients with Sepsis

Sepsis-3 criteria	*N* (%)
qSOFA	
1	17 (18)
3	75 (82)
SIRS	
2	18 (20)
4	74 (80)
Septic Shock	
No	73 (79)
Yes	19 (21)
Lactate	
Median (IQR)	2.9 (2.7–3.0)
Creatinine	
Median (IQR)	1.5 (1.6–1.7)
Albumin levels	
≥ 3.5	38 (41)
< 3.5	54 (59)
Beta2MG	
ISS	
I	20 (22)
II	35 (38)
III	37 (40)
R-ISS	
I	16 (17)
II	10 (11)
III	66 (72)
Frailty IMWG	
Frail	48 (52)
Non—frail	44 (48)
Renal Failure	
No	37 (40)
Yes	55 (60)
Anemia	
No	27 (29)
Yes	65 (71)
Immunoparesis	
No	28 (30)
Yes	64 (70)
Bone Disease	
No	16 (17)
Yes	76 (83)
Pathogen	
Fungal	18 (20)
Gram-negative	26 (28)
Gram-positive	21 (23)
Listeria Monocytogenes	9 (10)
Non isolated	18 (19)
Length of Hospitalization (days)	
Median (IQR)	14 (12–14)

*qSOFA*, Quick SOFA Score for Sepsis; *SIRS*, Systemic Inflammatory Response Syndrome; *KS*, Karnofsky Performance Status Scale; *Beta2MG*, Beta-2 Microglobulina; *ISS*, Multiple Myeloma International Staging System; *R-ISS*, Revised Multiple Myeloma International Staging System; *IMWG*, International Myeloma Working Group; *IQR*, Interquartile range

Among the MM patient population, a considerable number of individuals, 60% precisely, presented with renal failure at the time of sepsis diagnosis, underscoring the vulnerability of the renal system in these patients and the impact of sepsis on their renal function (Table 2). Anemia, defined by a hemoglobin level below 10 g/dL, was observed in 71% of the cases, reflecting the hematological disturbances accompanying sepsis in MM patients (Table 2).

The presence of bone disease, as defined by the CRAB criteria (hypercalcemia, renal insufficiency, anemia, and lytic bone lesions), was prevalent in 83% of the patients, with lytic bone lesions being the most common manifestation (Table 2). Additionally, immunoparesis, characterized by a decrease in one or more immunoglobulin classes, was observed in 70% of the patients [21].

### Microbiological analysis

Microbiological analysis yielded valuable insights into the underlying pathogens responsible for sepsis in these MM patients. Respiratory tract infections accounted for a significant portion, constituting 40% of the cases. Within this group, gram-negative bacteria such as *Klebsiella pneumoniae* and *Pseudomonas aeruginosa* were found to be predominant causative agents, as they were reported in 28% of the population. Gram-positive bacteria, including *Staphylococcus aureus* and *Streptococcus pneumoniae*, were responsible for 23% of the infections, indicating their substantial contribution to the overall septic burden. Additionally, fungal infections [22], predominantly caused by *Candida species (C. albicans, C. glabrata, C. tropicalis, C. parapsilosis*), were identified in 20% of the cases, highlighting the opportunistic nature of these pathogens in immunocompromised individuals [23]. The analysis revealed the distribution of different types of infections and the prevalence of specific pathogens (Table 2). Notably, *Listeria monocytogenes* infections were detected in 9.78% of the cases, primarily occurring in patients receiving CD38-directed antibody therapy (78% vs. 30%, *p* = 0.004), which warrants attention in the management of these patients (Table 3).

**Table 3:** Microbiological Analysis of *Listeria Monocytogenes* infection

	*Listeria monocytogenes*		
	No (*n* = 83)	Yes (*n* = 9)	*p-value
DARA-treatment, *n*(%)			
No	58 (70)	2 (22)	0.004
Yes	25 (30)	7 (78)	

^*^p-value (significance level ≤ 0.05)

Regarding the antimicrobial therapy, patients were treated as in Supplementary Table 3, following our Institutional and international Guidelines [24, 25] (Supplementary Table 4). Antimicrobial de-escalation was deemed essential upon the reporting of culture test results. Collaboration with infectious disease consultants or the sepsis team was employed to streamline therapy based on the identified pathogen and its antimicrobial sensitivity profile, along with clinical response and laboratory trends. Caution was advised regarding carbapenem use, reserved for essential cases, and considering substitution of vancomycin, teicoplanin, and daptomycin with cefazolin or oxacillin if methicillin-sensitive staphylococcus were isolated. The duration of antibiotic therapy was also selected by clinical progress and biomarker trends. Specifically, the antimicrobial therapies, matching the given schedule and posology (Supplementary Table 5), lasting longer than 7–10 days were deemed unnecessary, except in specific scenarios like slow clinical improvement.

### Univariate and cox-multivariate analysis

**Fig. 1:**
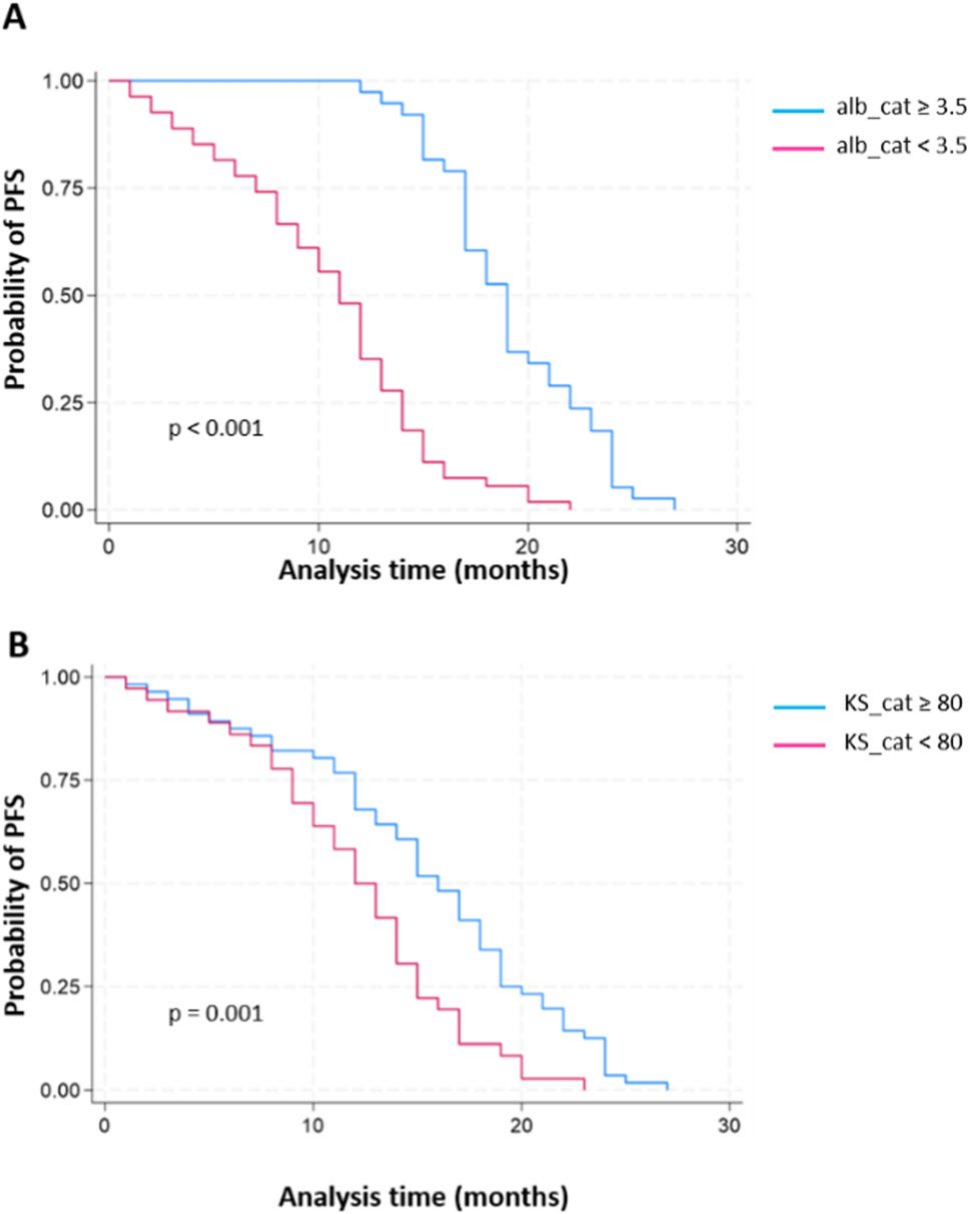
Kaplan–Meier estimates of PFS based on Albumin levels (**A**) and Karnofsky Performance Status Scale (**B**)

**Fig. 2:**
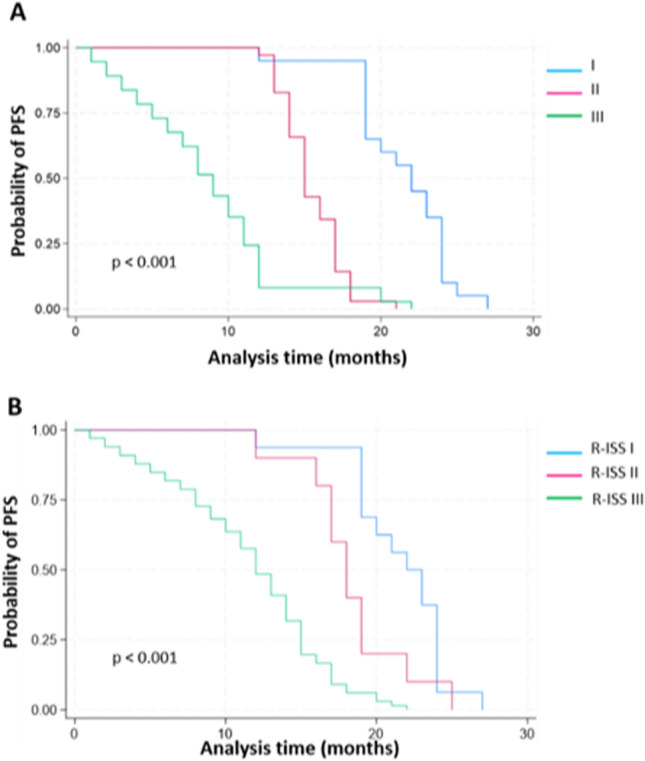
Kaplan–Meier estimates of PFS based on disease stages according to the International Staging System (ISS) (**A**) and the Revised International Staging System (R-ISS) (**B**)

PFS was deemed significantly longer in patients with albumin levels ≥ 3.5 than in patients with albumin levels < 3.5 (Log-rank test = 52.10, *p* < 0.001) and in patients with KS ≥ 80 vs < 80 (Log-rank test = 10.86, *p* = 0.001) (Fig. 1A and 1B). Moreover, PFS showed significant improvement among patients in earlier disease stages of MM, particularly those identified as ISS–I (Log-rank test = 73.27, *p* < 0.001) and R-ISS–I (Log-rank test = 45.19, *p* < 0.001), compared to others affected by more progressed forms of the underlying disease (Fig. 2A and B).

Cox univariate analyses of progression-free survival (PFS) showed statistically significant HR in patients with SIRS 4 vs 2 (HR = 0.56, *p* = 0.037), albumin levels < 3.5 vs $\ge$ 3.5 (HR = 5.04, *p* < 0.001), Karnofsky Performance Status Scale (KS) < 80 vs $\ge$ 80 (HR = 2.01, *p* = 0.002), age (HR = 1.02, *p* = 0.008) and late-stage vs early-stage disease according to International Staging System (ISS) (HR = 4.76 and HR = 12.52, both *p* < 0.001) and Revised-International Staging System (R-ISS) (R-ISS III vs R-ISS I, HR = 7.38, *p* < 0.001) (Table 4). The multivariate model confirmed the results for age (HR = 1.02, *p* = 0.005) and R-ISS III vs R-ISS I, HR = 7.08, *p* < 0.001).

**Table 4:** Univariate Analysis and Cox-Multivariate analysis of MM Patients with Sepsis

	Univariate analysis		Cox-multivariate	
	HR (95% CI)	*p*-value	HR (95% CI)	*p*-value
qSOFA				
1	1		1	
3	0.58 (0.33–1.01)	0.054	1.47 (0.22–9.74)	0.688
SIRS				
2	1		1	
4	0.56 (0.33–0.96)	0.037	0.51 (0.08–3.28)	0.484
Septic shock				
No	1		1	
Yes	0.71 (0.42–1.22)	0.222	0.79 (0.45–1.39)	0.419
KS				
≥ 80	1		1	
< 80	2.01 (1.29–3.14)	0.002	1.47 (0.92–2.33)	0.100
Age	1.02 (1.00–1.04)	0.008	1.02 (1.00–1.04)	0.005
Sex				
F	1		1	
M	0.90 (0.59–1.37)	0.646	1.11 (0.71–1.73)	0.621
Lactate	1.74 (0.47–6.45)	0.403		
Creatinine	1.88 (0.30–11.68)	0.494		
Albumin levels				
≥ 3.5	1			
< 3.5	5.04 (3.09–8.23)	< 0.001		
Beta2MG	3.13 (2.30–4.75)	< 0.001		
Cytogenetic risk				
No	1			
Yes	1.12 (0.74–1.70)	0.574		
ISS				
I	1			
II	4.76 (2.28–9.93)	< 0.001		
III	12.52 (6.18–25.32)	< 0.001		
R-ISS				
I	1			
II	2.03 (0.89–4.65)	0.092	2.01 (0.84–4.78)	0.112
III	7.38 (3.68–14.78)	< 0.001	7.08 (3.32–15.10)	< 0.001
Frailty IMWG				
Non—frail	1			
Frail	1.08 (0.71–1.63	0.698	1.26 (0.82–1.93)	0.284
Renal failure				
No	1			
Yes	0.73 (0.47–1.11)	0.148		
Anemia				
No	1			
Yes	0.69 (0.44–1.10)	0.128		
Bone Di sease				
No	1			
Yes	0.72 (0.41–1.24)	0.242		
Response				
Optimal	1			
Suboptimal	1.21 (0.80–1.83)	0.358		

*p*-value (significance level ≤ 0.05)

*qSOFA*, Quick SOFA Score for Sepsis; *SIRS*, Systemic Inflammatory Response Syndrome; *KS*, Karnofsky Performance Status Scale; *Beta2MG*, Beta-2 Microglobulina; *ISS*, Multiple Myeloma International Staging System; *R-ISS*, Revised Multiple Myeloma International Staging System; *IMWG*, International Myeloma Working Group; *HR*, Hazard Ratio; 95% CI Confidence Interval 95%

**Fig. 3:**
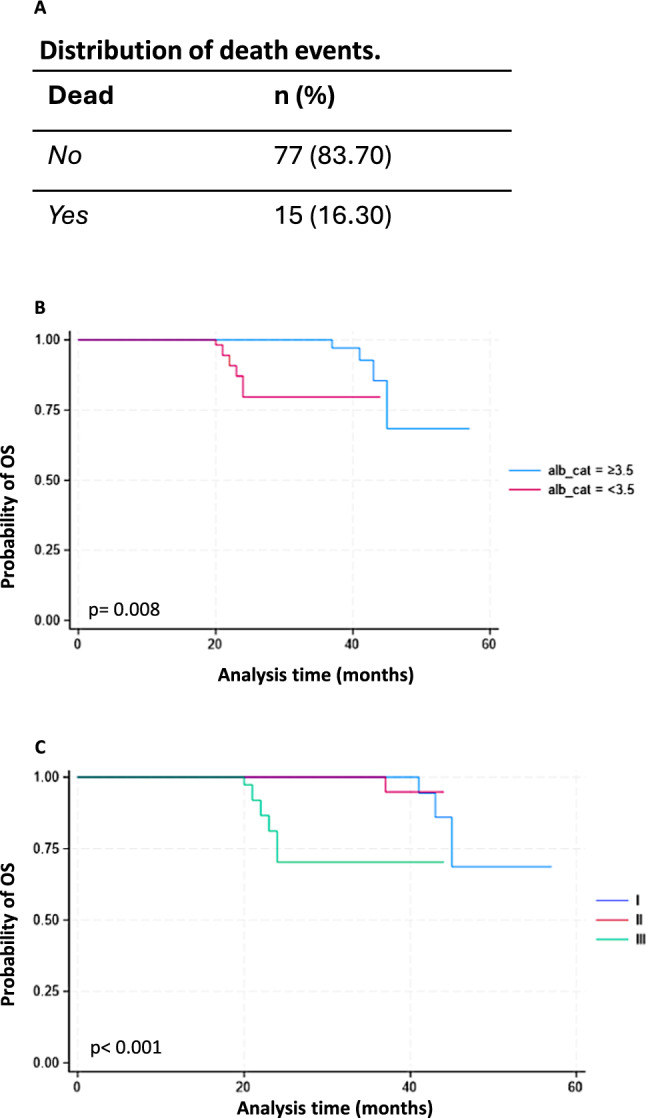
Distribution of death events (**A**) and Kaplan–Meier estimates of OS based on Albumin levels (**B**) and disease stages according to the International Staging System (ISS) (C)

Ancillary to the main outcomes, death events were recorded and summarized in Fig. 3A. While we do not have specific data on sepsis-related mortality, our study provides an overview of death events observed in our MM cohort during the study period. Statistically powered perspective studies will aim to include specific data on sepsis-related mortality. While acknowledging that the analysis of overall survival (OS) is beyond the scope of this manuscript, given the actual median survival of myeloma patients, the OS since the diagnosis of multiple myeloma to the death event was performed by applying the Kaplan–Meier estimator. We analyzed the course of the survival to the event weighted by explanatory variables (qSOFA = 1 and qSOFA = 3, anemia, renal failure, bone disease, immunoparesis, cytogenetics, frailty, response, karnofsky, R-ISS, albumin, ISS). OS was significantly different in patients with albumin levels ≥ 3.5 than in patients with albumin levels < 3.5 (Log-rank test = 6.92, *p* = 0.008) and among patients in earlier disease stages of MM, particularly those identified as ISS–I (Log-rank test = 16.94, *p* < 0.001), compared to others affected by more progressed forms of the underlying disease (Fig. 3B and C).

## Discussion

The present study conducted a retrospective analysis utilizing medical records from 92 NDMM patients who developed sepsis or septic shock. This comprehensive investigation aimed to elucidate various aspects of this patient population, including clinical characteristics, outcomes, immune profiling, microbiological data, and treatment modalities. By integrating sepsis scoring systems, such as qSOFA and SIRS, and evaluating important MM-related factors like International Staging System (ISS) staging, CRAB criteria [18], the 60% plasma cells, SLiM criteria, immunoparesis, and treatment approaches, including ASCT vs. non-ASCT, triplet regimens, and antibody therapies, including anti-CD38, a comprehensive understanding of the complex interplay between sepsis and MM can be achieved. Consistent with previous research, our findings highlight the heightened susceptibility of MM patients to sepsis, particularly those with advanced disease, compromised renal function, anemia, bone involvement, and immunoparesis. The identification of Listeria monocytogenes infections in MM patients receiving CD38-directed antibody therapy has already been described in previous literature [10]. It may be partially explained by delving into CD38’s roles during inflammatory-immune responses [26]. In particular, inflammatory stimuli-triggered up-regulated CD38 expression on neutrophils and macrophages’ membranes, responsible for regulating the complex mechanisms of endothelial transmigration (possibly via interaction with CD31) and contribute to the chemotactic recruitment of inflammatory cells towards peripheral sites of infection, may hint at a possible hindrance in these functions were it to be a depletion or inhibition of said receptor protein, as it has already been observed in murine models CD38 knocked out, which showed an increased susceptibility both to *S. pneumonia* and *L. monocitogenes* infections [27], which may be a result of inflammatory cells’ migration patterns alteration. Moreover, other than its role as a transmembrane receptor protein, CD38 has been found to have also enzymatic activities, in particular through the synthesis of Ca2⁺-mobilizing intracellular mediators, and subsequent activation of signaling pathways leading to chemokine receptors up-regulation (CXCR4, CCR7, N-formyl peptide receptor 1), cytoskeleton activation, adhesion molecules induction and even phagosomes maturation, therefore resulting in a CD38 depletion related impaired macrophage phagocytosis as well [28]. Considering the role of CD38 in directing the innate immune response against infective pathogens, a decrease in its functioning as a result of direct inhibitors’ effects (e.g. Daratumumab) could potentially lead to a higher risk of complicated infections in cohorts of already multifactorial immunoparetic MM patients, therefore underscoring the need for increased vigilance and the implementation of appropriate prophylactic measures in this specific subgroup.

Moreover, our study revealed significant associations between sepsis scores (qSOFA and SIRS) and clinical outcomes. Patients with higher sepsis scores exhibited elevated mortality rates and longer hospital stays, indicating a greater disease burden. Furthermore, higher sepsis scores were correlated with increased organ dysfunction, as evidenced by elevated levels of lactate and creatinine. These findings highlight the clinical relevance of sepsis scoring systems in assessing disease severity and predicting outcomes in MM patients. Regarding the MM-specific characteristics, a substantial proportion of patients presented with renal failure at the time of sepsis diagnosis, underscoring the importance of monitoring renal function and implementing appropriate interventions in this population. Anemia, defined as a hemoglobin level below 10 g/dL, was observed in most cases, reflecting the impact of MM on red blood cell production. The presence of bone disease according to the CRAB criteria was a prevalent feature, with lytic lesions being the most common manifestation. Immunoparesis, within an immunosuppressive immune-microenvironment, characterized by a decrease in one or more immunoglobulin classes, particularly affecting the IgG and IgA subclasses, further contributes to the immunocompromised state of MM patients and their vulnerability to infectious complications [23]. These findings provide insights into the contemporary treatment landscape for MM patients with sepsis and underscore the importance of individualized treatment strategies based on patient characteristics and disease status. Importantly, our study paves the way for additional prophylactic measures against pathogens such as Listeria monocytogenes and improving management strategies for sepsis in MM patients. Despite the valuable insights provided by this study, certain limitations should be acknowledged. The retrospective nature of the analysis introduces inherent limitations, including the reliance on available medical records and the potential for incomplete or missing data. The single-center design further restricts the generalizability of the findings, as patient populations and practices may vary across different healthcare settings. As both as out- and in-patient myeloma clinic, we acknowledge the marginal overestimation of sepsis incidence. Additionally, the relatively small sample size of the study cohort might limit the statistical power and generalizability of the results (including predisposing factors to increased mortality) and the short follow-up time does not allow proper evaluation of MM OS as by guidelines [20]. Recent robust evidence sketching frailty and immunoparesis pinpoint the need for a multimodal dynamic patient evaluation [29, 30]. Therefore, prospective multicenter studies with larger patient cohorts are warranted to validate these findings, provide more robust evidence, and further elucidate the optimal management strategies for sepsis in MM patients.

## Conclusions

This analysis of 92 newly diagnosed MM patients with sepsis or septic shock uncovers higher SIRS score in myeloma patients to be associated with worse PFS. The integration of sepsis scoring systems and consideration of important MM-related factors aid in risk stratification, guiding therapeutic decisions, and improving overall outcomes in MM patients with sepsis. Further research is necessary to optimize the management of sepsis in this population, including the prevention and management of immunoparesis, infections, also due to Listeria monocytogenes, and, ultimately, to improve patient outcomes.

## Supplementary Information

Below is the link to the electronic supplementary material.

Supplementary file1 (DOCX 16 KB)

Supplementary file2 (DOCX 16 KB)

Supplementary file3 (DOCX 15 KB)

Supplementary file4 (DOCX 16 KB)

Supplementary file5 (DOCX 24 KB)

## Data Availability

No datasets were generated or analysed during the current study.

## References

[1] Mateos MV, San Miguel JF. Management of multiple myeloma in the newly diagnosed patient. Hematol Am Soc Hematol Educ Program. 2017;2017(1):498–507. 10.1182/asheducation-2017.1.498 PMC6142596 29222298

[2] de la Rubia J, Cejalvo MJ, Ribas P. Infectious complications in patients with newly diagnosed multiple myeloma: a complication from the past? Leuk Lymphoma. 2016;57(2):258–68. 26428053 10.3109/10428194.2015.1088647

[3] Palumbo A, Anderson K. Multiple myeloma. N Engl J Med. 2011;364(11):1046–60. 21410373 10.1056/NEJMra1011442

[4] Russell BM, Avigan DE. Immune dysregulation in multiple myeloma: the current and future role of cell-based immunotherapy. Int J Hematol. 2023;117(5):652–9. 36964840 10.1007/s12185-023-03579-x PMC10039687

[5] Sørrig R, Klausen TW, Salomo M, Vangsted A, Gimsing P. Risk factors for infections in newly diagnosed Multiple Myeloma patients: a Danish retrospective nationwide cohort study. Eur J Haematol. 2019;102(2):182–90. 30485563 10.1111/ejh.13190

[6] Singer M, Deutschman CS, Seymour CW, et al. The third international consensus definitions for sepsis and septic shock (Sepsis-3). JAMA. 2016;315(8):801–10. 26903338 10.1001/jama.2016.0287 PMC4968574

[7] Chicca IJ, Heaney JL, Iqbal G, et al. Stratifying risk of infection and response to therapy in patients with myeloma: a prognostic study. NIHR J Libr. 2020. 10.3310/eme07100. 33301281

[8] Lin C, Shen H, Zhou S, et al. Assessment of infection in newly diagnosed multiple myeloma patients: risk factors and main characteristics. BMC Infect Dis. 2020;20(1):699. 32972385 10.1186/s12879-020-05412-w PMC7517606

[9] Cowan AJ, Green DJ, Kwok M, et al. Diagnosis and management of multiple myeloma: a review. JAMA. 2022;327(5):464–77. 35103762 10.1001/jama.2022.0003

[10] Khan S, Vaisman A, Hota SS, et al. Listeria susceptibility in patients with multiple myeloma receiving daratumumab-based therapy. JAMA Oncol. 2020;6(2):293–4. 31774462 10.1001/jamaoncol.2019.5098 PMC6902180

[11] Vincent JL, Moreno R, Takala J, et al. The SOFA (Sepsis-related Organ Failure Assessment) score to describe organ dysfunction/failure. On behalf of the working group on sepsis-related problems of the European Society of Intensive Care Medicine. Intensive Care Med. 1996;22(7):707–10. 8844239 10.1007/BF01709751

[12] Simpson SQ. SIRS in the time of sepsis-3. Chest. 2018;153(1):34–8. 29037526 10.1016/j.chest.2017.10.006

[13] Kumar SK, Rajkumar V, Kyle RA, et al. Multiple myeloma. Nat Rev Dis Primer. 2017;3:17046. 10.1038/nrdp.2017.46 28726797

[14] Teh BW, Harrison SJ, Slavin MA, Worth LJ. Epidemiology of bloodstream infections in patients with myeloma receiving current era therapy. Eur J Haematol. 2017;98(2):149–53. 27717026 10.1111/ejh.12813

[15] Offidani M, Corvatta L, Morè S, et al. Daratumumab for the management of newly diagnosed and relapsed/refractory multiple myeloma: current and emerging treatments. Front Oncol. 2020;10: 624661. 33680948 10.3389/fonc.2020.624661 PMC7928404

[16] Raje NS, Anaissie E, Kumar SK, et al. Consensus guidelines and recommendations for infection prevention in multiple myeloma: a report from the International Myeloma Working Group. Lancet Haematol. 2022;9(2):e143–61. 35114152 10.1016/S2352-3026(21)00283-0

[17] Lim C, Sinha P, Harrison SJ, Quach H, Slavin MA, Teh BW. Epidemiology and risks of infections in patients with multiple myeloma managed with new generation therapies. Clin Lymphoma Myeloma Leuk. 2021;21(7):444-450.e3. 33722538 10.1016/j.clml.2021.02.002

[18] Rajkumar SV, Dimopoulos MA, Palumbo A, et al. International myeloma working group updated criteria for the diagnosis of multiple myeloma. Lancet Oncol. 2014;15(12):e538-548. 25439696 10.1016/S1470-2045(14)70442-5

[19] Davies F, Rifkin R, Costello C, et al. Real-world comparative effectiveness of triplets containing bortezomib (B), carfilzomib (C), daratumumab (D), or ixazomib (I) in relapsed/refractory multiple myeloma (RRMM) in the US. Ann Hematol. 2021;100(9):2325–37. 33970288 10.1007/s00277-021-04534-8 PMC8357697

[20] Moreau P, San Miguel J, Sonneveld P, et al. Multiple myeloma: ESMO Clinical Practice Guidelines for diagnosis, treatment and follow-up. Annals Oncol. 2017;28:iv52–61. 10.1093/annonc/mdx096. 10.1093/annonc/mdx096 28453614

[21] Geng C, Yang G, Wang H, et al. Deep and partial immunoparesis is a poor prognostic factor for newly diagnosed multiple myeloma patients. Leuk Lymphoma. 2021;62(4):883–90. 33275060 10.1080/10428194.2020.1855345

[22] Teh BW, Teng JC, Urbancic K, et al. Invasive fungal infections in patients with multiple myeloma: a multi-center study in the era of novel myeloma therapies. Haematologica. 2015;100(1):e28-31. 25304609 10.3324/haematol.2014.114025 PMC4281332

[23] Vacca A, Melaccio A, Sportelli A, et al. Subcutaneous immunoglobulins in patients with multiple myeloma and secondary hypogammaglobulinemia: a randomized trial. Clin Immunol. 2018;191:110–5. 29191714 10.1016/j.clim.2017.11.014

[24] PDTA per l’Identificazione Precoce e la Gestione Tempestiva della Sepsi nel paziente adulto. In: https://www.sanita.puglia.it/documents/36067/434549/delibera+1158-2020+All.1.pdf/47b2f3bf-42fd-407c-a5f6-997fabf411ae. Accessed 20 December 2023.

[25] Evans L, Rhodes A, Alhazzani W, et al. Surviving sepsis campaign: international guidelines for management of sepsis and septic shock 2021. Crit Care Med. 2021;49(11):e1063–143. 10.1097/CCM.0000000000005337. 34605781 10.1097/CCM.0000000000005337

[26] Piedra-Quintero ZL, Wilson Z, Nava P, Guerau-de-Arellano M. CD38: an immunomodulatory molecule in inflammation and autoimmunity. Front Immunol. 2020. 10.3389/fimmu.2020.597959. 33329591 10.3389/fimmu.2020.597959 PMC7734206

[27] Lischke T, Heesch K, Schumacher V, Schneider M, Haag F, Koch-Nolte F, Mittrücker HW. CD38 controls the innate immune response against listeria monocytogenes. Infect Immun. 2013;81(11):4091–9. 10.1128/IAI.00340-13. 23980105 10.1128/IAI.00340-13 PMC3811837

[28] Partida-Sánchez S, Randall TD, Lund FE. Innate immunity is regulated by CD38, an ecto-enzyme with ADP-ribosyl cyclase activity. Microbes Infect. 2003;5(1):49–58. 10.1016/S1286-4579(02)00055-2. 12593973 10.1016/s1286-4579(02)00055-2

[29] Cook G, Pawlyn C, Cairns DA, Jackson GH. Defining FiTNEss for treatment for multiple myeloma. Lancet Healthy Longev. 2022;3(11):e729–30. 10.1016/S2666-7568(22)00218-5. 36356621 10.1016/S2666-7568(22)00218-5

[30] Sørrig R, Klausen TW, Salomo M, et al. Immunoparesis in newly diagnosed Multiple Myeloma patients: effects on overall survival and progression free survival in the Danish population. PLoS ONE. 2017;12(12):e0188988. 10.1371/journal.pone.0188988. 29216227 10.1371/journal.pone.0188988 PMC5720701

