# Supplementary material for: Comprehensive analysis of clinical outcomes, infectious complications and microbiological data in newly diagnosed multiple myeloma patients: a retrospective observational study of 92 subjects
Source: Clin Exp Med. 2024 Jun 27;24(1):137. doi: 10.1007/s10238-024-01411-2 (PMC11211138; doi:10.1007/s10238-024-01411-2)
Supplement: Supplementary file 1 — Supplementary file1 (DOCX 16 KB) [file 10238_2024_1411_MOESM1_ESM.docx]

**Supplementary Table 1. Median time (days) elapsed from treatment initiation and the sepsis event (variable “time interval”)**

|  | Time interval | **p-value |
| --- | --- | --- |
| qSofa  *1*  *3* | -30(-212;-21) -29(-311;-21) | 0.954 |
| SIRS  *2*  *3* | -35 (-179;-21)  -28.5 (-331;-21) | 0.843 |
| First line  *ASCT recipient*  *non-ASCT recipient* | -26 (-176;-17)  -37(-351;-21) | 0.068 |

*Data are expressed as median (IQR); **p-value for Wilcoxon rank-sum (Mann–Whitney) test
