# Supplementary material for: Comprehensive analysis of clinical outcomes, infectious complications and microbiological data in newly diagnosed multiple myeloma patients: a retrospective observational study of 92 subjects
Source: Clin Exp Med. 2024 Jun 27;24(1):137. doi: 10.1007/s10238-024-01411-2 (PMC11211138; doi:10.1007/s10238-024-01411-2)
Supplement: Supplementary file 2 — Supplementary file2 (DOCX 16 KB) [file 10238_2024_1411_MOESM2_ESM.docx]

**Supplementary Table 2. Treatment regimens of enrolled patients.**

| Treatment Regimen | n (%) |
| --- | --- |
| *Dara-RD* | 13 (14.13) |
| *Dara-VMP* | 7 (7.61) |
| *Dara-VTD* | 12 (13.04) |
| *RD* | 13 (14.13) |
| *VCD* | 22 (23.91) |
| *VMP* | 18 (19.57) |
| *VTD* | 7 (7.61) |

*Data are expressed as N(%); Dara = Daratumumab, V = Bortezomib, T = Thalidomide R = Lenalidomide, M = Melphalan, D = Dexamethasone P = Prednisone
