# Supplementary material for: Comprehensive analysis of clinical outcomes, infectious complications and microbiological data in newly diagnosed multiple myeloma patients: a retrospective observational study of 92 subjects
Source: Clin Exp Med. 2024 Jun 27;24(1):137. doi: 10.1007/s10238-024-01411-2 (PMC11211138; doi:10.1007/s10238-024-01411-2)
Supplement: Supplementary file 3 — Supplementary file3 (DOCX 15 KB) [file 10238_2024_1411_MOESM3_ESM.docx]

**Supplementary Table 3. Antimicrobial regimens of enrolled patients.**

| **Antimicrobial therapy** | **n (%)** |
| --- | --- |
| *Piperacillin-tazobactam or Cefepime +*  *Vancomycin (or Linezolid or Daptomicin) ±*  *Amikacin* | 63 (68.5) |
| *Meropenem + Vancomycin (Linezolid or*  *Daptomycin) ± Amikacin* | 11 (12.0) |
| *Add Anidulafungin or Caspofungin or*  *Micafungin to the chosen regimen* | 18 (19.6) |
