# Supplementary material for: Comprehensive analysis of clinical outcomes, infectious complications and microbiological data in newly diagnosed multiple myeloma patients: a retrospective observational study of 92 subjects
Source: Clin Exp Med. 2024 Jun 27;24(1):137. doi: 10.1007/s10238-024-01411-2 (PMC11211138; doi:10.1007/s10238-024-01411-2)
Supplement: Supplementary file 4 — Supplementary file4 (DOCX 16 KB) [file 10238_2024_1411_MOESM4_ESM.docx]

**Supplementary table 4. Risk factors for “difficult” pathogens.**

| **Common risk factors for antibiotic multi-resistance** |  | Prolonged hospitalization, long-term care, or other healthcare facility stays in the last 3 months. |
| --- | --- | --- |
|  |  | Multiple cycles of systemic antibiotic therapy, surgeries, or other invasive procedures in the last 3 months. |
|  |  | Hemodialysis, peritoneal dialysis. |
|  |  | Indwelling bladder catheter or Central Venous Catheter (CVC) or Percutaneous Endoscopic Gastrostomy (PEG) placement within the last month. |
|  |  | Known recent colonization with Multi-Drug Resistant (MDR) bacteria. |
|  |  | Complicated diabetes mellitus. |
|  |  | Advanced age. |
|  |  | Alcoholism. |
|  |  | High incidence in the hospital ward (>30%). |
|  |  | Immunocompromisation***. |
| **ESBL-producing enterobacteria** |  | Previous colonization and/or infection (in the last 12 months). |
| **Klebsiella pneumoniae KPC** |  | Previous colonization and/or infection (in the last 12 months) or high incidence in the hospital ward (> 30%). |
| **Pseudomonas aeruginosa** |  | Previous colonization and/or infection (last 12 months) by P. aeruginosa. |
|  |  | Pulmonary structural alterations (bronchiectasis, severe COPD, cystic fibrosis, bronchial obstruction). |
| **Staphylococcus aureus resistant to methicillin (MRSA)** |  | Previous colonization and/or infection (last 12 months). |
|  |  | Presence of devices, endovascular and otherwise, in place for > 48 hours. |
|  |  | Hemodialysis, peritoneal dialysis. |
|  |  | IV drug addicts. |
|  |  | Pneumonia during or after influenza. |
|  |  | High incidence in the inpatient unit (>30%). |
| **Candida spp.** |  | Recent multifocal infection or colonization with Candida spp. |
|  |  | Major abdominal surgery/necrotizing pancreatitis. |
|  |  | Parenteral nutrition. |
|  |  | CVC or PEG in place for > 48 hours. |

****Immunocompromisation: HIV with CD4+ < 200/mm^3^, marrow and/or solid organ transplant,* *chemotherapy in the last 2 months, steroid therapy (prednisone 20 mg/day or equivalent) for at least 4 weeks, persistent neutropenia (< 500 mm^3^), splenectomy.*
