# Supplementary material for: Comprehensive analysis of clinical outcomes, infectious complications and microbiological data in newly diagnosed multiple myeloma patients: a retrospective observational study of 92 subjects
Source: Clin Exp Med. 2024 Jun 27;24(1):137. doi: 10.1007/s10238-024-01411-2 (PMC11211138; doi:10.1007/s10238-024-01411-2)
Supplement: Supplementary file 5 — Supplementary file5 (DOCX 24 KB) [file 10238_2024_1411_MOESM5_ESM.docx]

**Supplementary Table 5. Empiric antimicrobial therapy and related posology [24, 25].**

| **Clinical Severity/MDR (Multidrug resistance) risk** | | | | | | | |  | | | | **First line treatment** | | | | | | | | | | |
| --- | --- | --- | --- | --- | --- | --- | --- | --- | --- | --- | --- | --- | --- | --- | --- | --- | --- | --- | --- | --- | --- | --- |
| **Sepsis** | | | | | | | |  | | | | Piperacillin-tazobactam *or* Cefepime + Vancomycin (*or* Linezolid *or* Daptomycin) ± Amikacin+ | | | | | | | | | | |
| **Allergy to beta-lactams *or* risk of multi-resistant bacteria *or* Septic shock** | | | | | | | |  |  |  |  | Meropenem + Vancomycin (*or* Linezolid *or* Daptomycin) ± Amikacin | | | | | | | | | | |
| **Sepsis/septic shock and risk of invasive candidiasis** | | | | | | | |  |  |  |  | Addition of Anidulafungin *or* Caspofungin *or* Micafungin to the chosen regimen | | | | | | | | | | |
| **Posologic recommendations** | | | | | | | | | | | | | | | | | | | | | | |
|  | **Normal Renal function** | **Creatinine Clearance (ml/min)** | | | | | | | | | | | | | | | | | | | **Renal replacement therapy** | |
|  |  | **> 50-90** | | | | | | **10-50** | | | | | | | | **< 10** | | | | | **IHD** | **CRRT** |
| **Piperacillin-tazobactam** | ^a^ LD: 4.5 g  MD: 4.5 g/6 h ^b^ | ^a^ LD: 4.5 g  MD: 4.5  g/6 h ^b^ | | | | | | ^a^ LD: 4.5 g  MD: 4.5  g/8 h ^b^ | | | | | | | | ^a^ LD: 4.5 g  MD: 2.25  g/6 h ^b^ | | | | | ^a^ LD: 4.5 g  MD: 2.25  g/8 h ^b^  +  750 mg post-dialysis | ^a^ LD: 4.5 g  MD: 2.25-4.5  g/8 h ^b^ |
|  | **Normal Renal function** | **Creatinine Clearance (ml/min)** | | | | | | | | | | | | | | | | | | | **Renal replacement therapy** | |
|  |  | **> 60-90** | | | | | **30-60** | | | | **11-29** | | | | | | | | **< 11** | | **IHD** | **CRRT** |
| **Cefepime** | IA: 2 g/8-12 h  CI ^c^: 6 g/24 h | IA: 2 g/8-12 h  CI ^c^: 6 g/24 h | | | | | IA: 2 g/12 h  CI ^c^: 4 g/24 h | | | | IA: 1-2 g/24 h  CI ^c^: 2 g/24 h | | | | | | | | IA: 500 mg – 1 g/24 h | | IA: 1 g/24 h post-dialysis | IA: 2 g/24 h |
|  | **Normal Renal function** | **Creatinine Clearance (ml/min)** | | | | | | | | | | | | | | | | | | | **Renal replacement therapy** | |
|  |  | **> 50-90** | | | | | | **10-50** | | | | | | | | **< 10** | | | | | **IHD** | **CRRT** |
| **Meropenem** | 3-6 g/6-8 h ^b^ | 1 g/8 h ^b^ | | | | | | ^a^ LD: 2 g  MD: 500 mg-1 g/12 h ^b^ | | | | | | | | ^a^ LD: 1-2 g  MD: 500 mg-1 g/24 h ^b^ | | | | | ^a^ LD: 2 g  MD: 500 mg-1 g/24  h ^b^ post-dialysis | 1 g/12 h ^b^ |
|  | **Normal Renal function** | **Creatinine Clearance (ml/min)** | | | | | | | | | | | | | | | | | | | **Renal replacement therapy** | |
| **Amikacin**  (mg/kg) |  | **> 80** | | | | **60-80** | | | **40-60** | | | | | | **20-40** | | | | | **10-20** | **IHD** | **CRRT** |
| Single daily administration | 15-20/24 h | 15-20/24 h | | | | 15-20/36 h | | | 15-20/48 h | | | | | | 15-20/60 h | | | | | 15-20/72 h | 15-20/96 h post-dialysis | 15-20/36-48 h |
| Divided daily-dose ^d^ | 7.5/12 h | 7.5/12 h | | | | 7.5/12 h | | | 7.5/24 h | | | | | | 4/24 h | | | | | 4/48 h | 3/72 h post-dialysis | 7.5/24 h |
|  | **Normal Renal function** | | | **Creatinine Clearance (ml/min)** | | | | | | | | | | | | | | | | | **Renal replacement therapy** | |
|  |  |  |  | **> 50-90** | | | | | | | | **10-50** | | | | | | **< 10** | | | **IHD** | **CRRT** |
| **Vancomycin** | ^a^ LD: 15-20 mg/kg  (25-30 mg/kg for septic shock)  MD:  CI: 30-40 mg/kg/die  IA: 7.5-10 mg/kg/6 h ^e^ | | | ^a^ LD: 15-20 mg/kg  (25-30 mg/kg for septic shock)  MD:  CI: 30-40 mg/kg/die  IA: 7.5-10 mg/kg/6 h ^e^ | | | | | | | | 500 mg/24-96 h | | | | | | 500 mg/48-96 h | | | 500 mg/48-96 h | 500 mg/6 h |
|  | **Normal Renal function** | | | **Creatinine Clearance (ml/min)** | | | | | | | | | | | | | | | | | **Renal replacement therapy** | |
|  |  |  |  | **> 30** | | | | | | | | | **< 30** | | | | | | | | **IHD** | **CRRT** |
| **Daptomycin** | 6-8 mg/kg/24 h | | | 6-8 mg/kg/24 h | | | | | | | | | 6 mg/kg/48 h | | | | | | | | 6 mg/kg/48 h,  9 mg/kg/72 h post-dialysis | 6 mg/kg/24 h |
|  | **Creatinine Clearance (ml/min)** | | | | | | | | | | | | | | | | | | | | **Renal replacement therapy** | |
|  | **> 50** | | | | **20-50** | | | | | | | | | **< 20** | | | | | | | **IHD** | **CRRT** |
| **Linezolid** | 600 mg/12 h | | | | Normal dose | | | | | | | | | Normal dose | | | | | | | Normal dose | Normal dose |
|  | **Normal Renal function** | | **Creatinine Clearance (ml/min)** | | | | | | | | | | | | | | | | | | **Renal replacement therapy** | |
|  |  |  | **> 50-90** | | | | | | | **10-50** | | | | | | | **< 10** | | | | **IHD** | **CRRT** |
| **Anidulafungin** | First dose: 200 mg  MD: 100 mg/24 h | | Normal dose | | | | | | | Normal dose | | | | | | | Normal dose | | | | Normal dose | Normal dose |
| **Caspofungin** | First dose: 70 mg  MD: 50 mg/24 h,  70 mg/24 h if BW > 80 kg | | Normal dose | | | | | | | Normal dose | | | | | | | Normal dose | | | | Normal dose | Normal dose |
| **Micafungin** | 100 mg/24 h | | Normal dose | | | | | | | Normal dose | | | | | | | Normal dose | | | | Normal dose | Normal dose |

*IHD, intermittent hemodialysis; CRRT, continuous renal replacement therapy; LD, loading dose; MD, maintenance dose; IA, intermittent administration; CI, continuous infusion; BW, body weight. a: loading dose must be administered immediately before initiating maintenance regimen; b: single dose duration of infusion is 4 hours;c: preceded by loading dose of 15 mg/kg over a 30 minutes period of administration;d: to be favored in case of: ascites, burns extension > 20% of total body surface, cystic fibrosis, endocarditis, pregnancy, severe renal failure (in the latter, alternative antibiotics should be opted for preferably);e: 2 g maximum for each dose.*
